# Implementation of Patient-Reported Outcome Measures in Primary Care: Challenges and Future Directions in Saudi Arabia

**DOI:** 10.7759/cureus.102686

**Published:** 2026-01-31

**Authors:** Mohammad Shibly Khan, Mamdouh Falih Althaqeel, Elmuez Eltayeb Elnaiem, Manal A Elimam, Isameldin Abdelrahim, Omar Abdulrahman Alayed, Talal Elfadil Mahdi, Fayez Salem Alenzi, Abdurhman Saad Alsuliman, Mesfer Ahmed Alamri, Shibli Sayeed, Amirah Musaad Alkhurayji

**Affiliations:** 1 Health Excellence, King Salman Hospital, Riyadh First Health Cluster, Riyadh, SAU; 2 Hospital Administration, King Salman Hospital, Riyadh First Health Cluster, Riyadh, SAU; 3 Quality and Patient Safety, King Salman Hospital, Riyadh First Health Cluster, Riyadh, SAU; 4 Directorate of Health Program and Chronic Disease, Ministry of Health, Riyadh, SAU; 5 Community Health Excellence, Riyadh First Health Cluster, Riyadh, SAU; 6 Population Health Management and Research Department, Riyadh First Health Cluster, Riyadh, SAU; 7 Academic Affairs, King Salman Hospital, Riyadh First Health Cluster, Riyadh, SAU; 8 Public Health, Ministry of Health, Riyadh, SAU; 9 Family Medicine, King Saud Bin Abdulaziz University for Health Sciences College of Medicine, Riyadh, SAU

**Keywords:** healthcare transformation, patient-centred care, patient reported outcome measures, primary care, saudi arabia, value-based care

## Abstract

Patient-reported outcome measures (PROMs) have become increasingly central to global health systems as they shift toward value-based, person-centered models of care. In Saudi Arabia, the Health Sector Transformation Program under Vision 2030 has created a national mandate for improving clinical outcomes, strengthening primary healthcare services, and enhancing patient experience. PROMs provide a systematic means of capturing patients’ perceptions of their symptoms, functional health, and quality of life, thereby delivering information that complements clinical assessments and supports shared decision-making. Despite their growing importance, the integration of PROMs into primary care settings across Saudi Arabia remains incomplete, and their implementation varies across health clusters. This review examines the rationale for PROM use, evaluates their applicability to Saudi primary care, discusses cultural and operational barriers, and outlines strategies for sustainable adoption within the evolving digital health ecosystem. The review contextualizes PROMs within national policies, emerging digital platforms, and the shifting landscape of chronic disease management in the Kingdom, concluding that PROMs have the potential to become a foundational element of Saudi Arabia’s value-based primary care model.

## Introduction and background

The healthcare sector in Saudi Arabia is undergoing major restructuring as part of Vision 2030’s Health Sector Transformation Program, which envisions the health system to be comprehensive, effective, and integrated through innovation, financial sustainability, and disease prevention while improving access to healthcare [1]. Improving the value of healthcare services is one of the major objectives of the transformation program [1]. While the transformation program aligns with the traditional notion of the primary health being the first portal of contact between the community and health care services, it seeks to transform the provision of existing primary care services by focusing on value-based care [2]. In the new health services delivery model, the services are to be provided by the accountable care organizations, referred to as Health Clusters, while the role of the Ministry of Health will be that of a super-regulator [2].

The health profile of Saudi Arabia is in a transition with an increasing burden of non-communicable diseases, which warrants the emphasis on rigorous implementation of preventive strategies while keeping the principles of patient-centered care in Dehradun [3]. The health system in Saudi Arabia connects the patients through primary health care centers, which makes the primary care services a strong pillar of health care service delivery [2].

Patient-reported outcome measures (PROMs), which collect patients’ evaluations of their own health status, have been recognized globally by organizations such as the World Health Organization (WHO) and the Organization for Economic Co-operation and Development (OECD) as essential components of patient-centered, value-based models of care [4,5]. In Saudi Arabia, implementing PROMs aligns directly with national goals of improving quality, transparency, and accountability in primary healthcare (PHC) services. Moreover, international bodies, such as the International Consortium for Health Outcomes Measurement (ICHOM), have promoted standardized PROM sets for major conditions, many of which are relevant to Saudi Arabia’s epidemiological profile [6].

Methodology

This review adopts a flexible narrative review approach, which is based on a literature search conducted during December 2025 in the PubMed and Google Scholar databases, along with reviewing the policy documents available through official websites. The search aimed to identify studies and documents related to the current status and strategies for PROM implementation in primary care in Saudi Arabia. The keywords used for searching the databases were "patient reported outcome measures", "primary care", "Saudi Arabia", "healthcare transformation", "vision-2030".

## Review

PROM within Vision 2030 and the health sector transformation

Saudi Arabia’s shift toward value-based healthcare reflects global trends emphasizing health outcomes over service volume. PROM is one of the main features of the Saudi health sector transformation strategy. The strategy was built based on the Saudi Vision 2030, which was launched in 2016 [1]. The Saudi Vision 2030 explicitly aims to create a vibrant society in which all citizens can thrive, emphasizing improvement in health, education, housing, entertainment, and opportunities for fulfilling lives [1]. Guided by a person-centric approach, Vision 2030 has shaped Saudi health policy towards comprehensive, integrated, and preventive services, leading to near-universal health coverage and better population health outcomes [1]. Within the health system, the people-(patient)-centered approach is grounded in a set of core principles, including respect for patients’ value, preferences, and expressed needs; coordination and integration of care; effective information sharing, communication, and education; shared decision-making and support for self-care; physical comfort; emotional support and the reduction of fear and anxiety; involvement of family and friends; and continuity of care with smooth transitions across services [7]. These principles are closely aligned with the key objectives of Saudi Arabia’s Health Sector Transformation Program (HSTP), which aims to improve the quality and efficiency of services, strengthen population health and prevention, and ensure financial sustainability [1,2]. The HSTP places strong emphasis on “beneficiary experience,” recognizing it as central to achieving Vision 2030 health goals by establishing an integrated and effective system that prioritizes individual well-being. This shift from provider-centered to beneficiary-centered care moves the health system from fragmented care toward holistic, patient-centered services enabled by digital integration, performance measurement, and standardized approaches to enhance satisfaction, quality, and access accessibility [1].

The new Model of Care (MoC) strategy in the Saudi health system further illustrates the strong emphasis placed by the Health Sector Transformation Program on a patient-centered approach [8]. The MoC translates patient-centeredness into practice by integrating it within organizational structures, care pathways, workflows, and performance indicators. Ultimately, a MoC can be regarded as effective only when it delivers genuinely patient-centered care in everyday practice, rather than merely reflecting this principle at the design stage [9]. Furthermore, the Saudi HSTP clearly demonstrates a patient-centered approach through active patient engagement in care planning and decision-making, the implementation of health education and promotion initiatives, strengthened community participation and accountability, and enhanced support for self-care and chronic disease management [10].

Patient-reported experience is already an integrated national activity within the Saudi health system, monitored through the National Centre for Patient Experience [11]. Chronic disease risk reduction is one of the priorities identified in the National Health Transformation Strategy [3], which has strong relevance to the patient-reported outcomes. Digital transformation, led by the Saudi Data and AI Authority (SDAIA) and the National Digital Transformation Unit, supports the integration of ePROMs into digital health platforms such as “Sehhaty” and “Mawid” [12].

Globally, countries with mature value-based systems, such as the UK (NHS), the Netherlands, and Sweden, routinely incorporate PROMs to evaluate functional outcomes, mental health, and quality of life [13,14]. This global evidence base supports the relevance of PROM to the Saudi context. WHO and the International Diabetes Federation emphasize the utility of PROMs in improving glycemic control, patient adherence, and early detection of diabetes-related distress [15]. Saudi Arabia’s high prevalence of diabetes, with an estimated adult prevalence of 23%, has strong justification for adopting diabetes-specific PROM [16].

Mental health has also gained national priority through initiatives such as the Saudi National Mental Health Policy and the integration of mental health screening in primary health care [17]. PROMs such as the Patient Health Questionnaire-9 (PHQ-9) and Generalized Anxiety Disorder 7-item (GAD-7) are validated in Arabic and widely used in Gulf countries [18]. For musculoskeletal diseases, especially knee and lower-back pain, validated PROMs, such as the Oxford Knee Score and Roland-Morris Disability Questionnaire, have been used and recommended by international orthopaedic societies [19]. General health measurement using EQ-5D also aligns with regional research efforts and has been successfully adapted in several Arabic-speaking populations [20].

PROMs can improve clinical communication, enhance care continuity, and support the early detection of deteriorating health status, consistent with evidence from WHO primary care studies [21]. In addition, PROM-based decision-making has been shown internationally to reduce unnecessary referrals, improve medication adherence, and strengthen shared decision-making [5].

At the health-system level, PROMs support the Kingdom’s transition toward value-based care by supplying measurable and comparable patient-centered outcomes [22]. PROMs also contribute to national-level strategic planning by identifying population health gaps, informing resource allocation, and supporting early prevention programs. Countries such as Denmark and the Netherlands have used national PROM datasets to shape PHC policies, and similar approaches could guide Saudi Arabia’s PHC transformation [23].

Challenges

Although the implementation of PROMs in Saudi Arabia is relatively recent and remains in an early developmental phase, international experience and early pilots provide valuable insight into the challenges that already exist or are likely to emerge as adoption scales up. Recognizing these challenges at an early stage is therefore essential, not as a critique of current efforts, but as a forward-looking risk-mitigation approach. By anticipating structural, technical, and governance-related barriers before PROMs become fully embedded into routine care and national reporting, Saudi Arabia has an opportunity to proactively address these issues, accelerate system readiness, and ensure that PROMs progress from isolated pilot tools into sustainable system-wide instruments that meaningfully inform patient-centered care, performance, accountability, and health policy decision-making. To provide a comprehensive and structured analysis of the challenges to PROMS implementation in Saudi Arabia, the WHO Health System Building Blocks framework is used as an analytical lens [24]. 

Governance

While PROMs are nationally promoted as a part of transition ward value-based and patient-centered care, uneven governance structures at the cluster level risk translating this strategic intent into fragmented and inconsistent practices [2,25]. Differences in delegated authority limit some clusters' ability to operationalize PROMs. Clusters with financial limitations or administrative autonomy may be unable to invest in digital PROM platforms, integrate PROM tools into electronic health records, or redesign clinical workflows to support routine data collection. Consequently, PROMs may remain confined to small-scale pilots rather than becoming embedded in routine care. A maturity-based, system-wide PROM implementation framework emphasizes that governance, standardization, and measurement infrastructure are essential for PROMs to support benchmarking and value-based purchasing [26].

Health information systems

The digital ecosystem in Saudi Arabia is undergoing a significant overhaul, as envisaged in the digital strategy adopted by the Ministry of Health [27]. On the other hand, health clusters are uneven, and a previous study illustrates the challenges of implementing large-scale electronic health records in primary care [28]. Data governance readiness poses a critical barrier to PROMs integration. Any cluster with weak data governance frameworks will struggle to standardize PROM instruments, ensure data quality, or link PROMs with clinical and utilization data. This results in parallel data systems, manual reporting processes, and delayed feedback to clinicians and managers, diminishing the perceived value of PROMs and increasing the reporting burden.

The barriers for electronic health registry (EHR) implementation range from organizational and operational obstacles, training and support gaps, workflow disruptions, and varying readiness, all of which are directly relevant to PROM digital integration. Further Saudi-based work evaluating EHR implementation phases reinforces the importance of preparedness, usability, and sustained support factors, which also determine whether PROMs become practice rather than intermittent data collection [28]. To facilitate significant system learning in primary care, the OECD's Paris project emphasizes the need to standardize and compare patient-reported data. PROMs risk becoming independent initiatives with little influence on policy in the absence of this standardization and harmonization [5].

A robust IT infrastructure, along with stakeholder engagement, with set standards, is the key requirement for the health system to implement the PROM across the system [29]. Lack of connectivity and technical support, in addition to frequent changes in staff, have been cited as the major obstacles in implementing large-scale electronic health records implementation in primary health care centers in Saudi Arabia [26].

Workforce

Primary care clinicians often perceive PROMs as an additional administrative burden unless the measures are linked to clear clinical decisions, e.g., stepped care pathways, medication adjustments, referral thresholds, and aligned with consultation flow. A qualitative article at the primary care level shows that clinicians may question the purpose of PROM collection, worry about time burden, and be concerned about reliability and potential constraints on consultations. They also expressed their struggle with interpretation when results do not translate into actionable steps, a problem that is amplified when feedback loops are weak [21].

Common barriers include competing clinical demands, uncertainty about how to use PROMs, and insufficient support (training, facilitation, workflow redesign). It is suggested that training should move beyond (how to administer PROMs) to "how to act on scores" clinical pathways, shared decision-making scripts, and escalation protocols [30]. Lack of scientific rigor among primary care physicians [31] could also be seen as a relative barrier for PROMs implementation in the wider perspective. On the other hand, trust in primary care physicians [32] could be a facilitator for its implementation. Workforce-related challenges include limited training in PROM interpretation and a lack of clear guidelines for PROM-based decision pathways [33].

Cultural acceptability

Cross-cultural adaptation is a crucial issue in implementing any tool. PROMs must demonstrate conceptual equivalence, acceptability, and measurement performance in both the target population and the clinical context. In Saudi Arabia, cultural norms may influence disclosure of emotional distress, functional limitations, or sensitive social concerns, potentially increasing non-response, especially for mental health-related PROMs [4].

While Arabic-language tools exist and have demonstrated psychometric validity in regional populations, local validation and implementation testing in primary care remain important, as measurement performance can vary by setting and population. For instance, Arabic versions of the PHQ instrument have been validated in Saudi samples, supporting feasibility for depression and anxiety screening-related PROM approaches [34]. Generic health status instruments (e.g., EQ-5D) have demonstrated validity and reliability in Arabic-speaking populations, but their implementation in Saudi primary care still requires attention to cultural interpretation and practical feasibility [20].

Validation of any tool in the Arabic language cannot guarantee workflow fluency. The implementers should prioritize instruments with evidence for Arabic psychometrics, clear interpretability, and feasible administration. Cultural norms may also influence patients’ willingness to report certain symptoms, particularly mental health issues, emotional distress, or social functioning [35].

Data use, performance, and unintended consequences

If clinicians believe PROM data will be used primarily for surveillance, penalties, or reimbursement without appropriate case-mix adjustment, engagement may decline, and data quality may deteriorate. Evidence on PROM feedback and performance data highlights the potential for unintended consequences when outcomes are publicly reported or used in performance management without careful design [36]. There is an emerging national direction toward PROM adoption in the private sector ecosystem, reinforcing the need to build trust, transparency, and safeguards around how PROM data will be interpreted and used [37].

All these challenges are interdependent, as governance gaps weaken standardization, limited digital maturity constrains feedback loops, poor workflow integration increases staff resistance, and cultural measurement issues undermine validity and comparability. Various experiences emphasize staged approaches, co-design with clinicians and patients, piloting with the right feedback cycles, and building facilitation capacity. It will be imperative to validate usability and clinical value before scaling [30].

Accordingly, Saudi primary care PROM implementation is likely to be most successful when introduced through clearly defined clinical use cases, e.g., diabetes distress, depression severity, embedded in routine workflow, supported by digital structure, and governed by transparent polices for data stewardship, risk adjustment, and appropriate use. Having dedicated administrative staff (at the local level) to manage PROM will also aid in successful implementation by reducing the burden on physicians.

Gaps in cultural adaptation and validation of PROMs have been reported to be the major constraints for PROM administration among Saudi patients [4]. Cross-cultural adaptation is essential, as Saudi patients may interpret concepts such as mental distress, pain disability, and social functioning differently from populations in Western countries.

Implementation strategies and the future of PROMs in Saudi Arabia

Successful PROM integration requires system-level coordination. WHO recommends national standard sets, digital integration, clinician training, and policy enforcement as core components of PROM implementation [4]. The global standard outcome sets can be aligned at the local level. Moreover, the standard tools have been validated in the Arabic language, which makes the usage more scientifically sound in terms of standardization [18,20]. Digital integration should follow national interoperability guidelines developed by the Saudi Health Council and the National Health Information Centre [38].

Clinician training can be integrated into postgraduate family medicine programs and continuing professional development, following WHO’s primary health care training models and international PHC PROM training methods [21,38]. Saudi Arabia’s rapid digitalization and adoption of artificial intelligence create opportunities for predictive analytics using PROM data. SDAIA’s national AI strategy emphasizes integrating patient-reported information into clinical decision-support systems [9]. PROMs could eventually support personalized care, automated triaging, and risk stratification. Countries such as Australia and Canada have used PROMs to drive reimbursement reforms, improve patient engagement, and enhance chronic disease management outcomes [39]. Saudi Arabia’s shift toward accountable care organizations mirrors these models, suggesting PROMs will soon play a central regulatory role.

Value-based purchasing is one of the major transformation initiatives [40], which involves payment reimbursed based on the quality and outcome of the care provided [41]. The development and implementation of PROM have been proposed as one of the enablers of value-based purchasing by the Council of Health Insurance (CHI) [37]. This puts the PROM at the forefront of the research landscape for future development and implementation strategy. At the same time, accountable care organizations have to implement this as one of the reportable performance indicators throughout the system [33].

## Conclusions

PROM implementation is one of the foundations for achieving the objective of value-based care under healthcare transformation in Saudi Arabia. Not only does it serve the core purpose of value-based care, but it has also been advocated as a strong tool for person-centered care. Although system-wide implementation of PROM is facing challenges spanning different domains, the existing policy framework supports its implementation.

Capacity building of the healthcare providers, along with stakeholder engagement, is the key to achieving the desired outcome. Unified electronic health records across the system is another facilitator for PROM implementation. More research and development efforts are needed to meet the felt need of PROM implementation at the primary care level.

## References

[1] (2025). Health Sector Transformation Program. Saudi Vision 2030. Kingdom of Saudi Arabia. https://www.vision2030.gov.sa/en/explore/programs/health-sector-transformation-program.

[2] Alasiri AA, Mohammed V (2022). Healthcare transformation in Saudi Arabia: an overview since the launch of Vision 2030. Health Serv Insights.

[3] World Bank Group (2021). Noncommunicable Diseases in Saudi Arabia: Toward Effective Interventions for Prevention. https://documents1.worldbank.org/curated/en/336261636951634235/pdf/Noncommunicable-Diseases-in-Saudi-Arabia-Toward-Effective-Interventions-for-Prevention.pdf.

[4] (2025). World Health Organization. Patient-reported experiences in primary care: metrics and assessment tool, rapid version. Geneva.

[5] (2025). Patient-Reported Indicator Surveys (PaRIS). Paris.

[6] (2019). International Consortium for Health Outcomes Measurement (ICHOM). Sets of patient-centered outcome measures. https://www.ichom.org/patient-centered-outcome-measures/.

[7] International Alliance of Patients' Organizations (2007). What is Patient-Centered Healthcare? A Review of Definitions and Principles. International Alliance of Patients' Organizations. London, UK. What is Patient-Centered Healthcare? A Review of Definitions and Principles.

[8] Chowdhury S, Mok D, Leenen L (2021). Transformation of health care and the new model of care in Saudi Arabia: Kingdom's Vision 2030. J Med Life.

[9] Cassidy S, Solvang ØS, Granja C, Solvoll T (2024). Flipping healthcare by including the patient perspective in integrated care pathway design: a scoping review. Int J Med Inform.

[10] Alshehri AA, Abduljawad AA (2025). Impact of the Saudi Health Sector Transformation Program (SHSTP): a mixed-methods evaluation of patient-centered care and digital health adoption. Healthcare (Basel).

[11] (2025). Patient experience measurement program. Ministry of Health. Kingdom of Saudi Arabia. https://www.moh.gov.sa/en/Ministry/pxmp/Pages/default.aspx.

[12] (2025). Saudi Data & AI Authority (SDAIA). National Strategy for Data & AI. https://sdaia.gov.sa/en/SDAIA/SdaiaStrategies/Pages/NationalStrategyForDataAndAI.aspx.

[13] (n.d). NHS England. PROMs programme annual report. https://www.england.nhs.uk/statistics/statistical-work-areas/proms/.

[14] Heintz E, Jacobsson G, Ernstsson O, Janssen B, Korkmaz S, Nilsson E (2022). Collection and use of EQ-5D in Swedish health care. Final report of the Swedish PROMs program. https://qrcstockholm.se/download/18.3bd89e09180474f0f6f2976e/1650868011792/Final-report-Swedish-PROMs-program.pdf.

[15] Alsubahi N, Groot W, Alzahrani AA, Pavlova M (2025). Patient-reported experience, patient-reported outcome and overall satisfaction with care: what matters most to people with diabetes?. Prim Care Diabetes.

[16] (2025). Statistical Year Book 2022: prevalence of non-communicable diseases. Ministry of Health. Kingdom of Saudi Arabia. Saudi Health Indicators.

[17] Qureshi NA, Al-Habeeb AA, Koenig HG (2013). Mental health system in Saudi Arabia: an overview. Neuropsychiatr Dis Treat.

[18] Alaqeel S, Alfakhri A, Alkherb Z, Almeshal N (2022). Patient-reported outcome measures in Arabic-speaking populations: a systematic review. Qual Life Res.

[19] Clement ND, Afzal I, Liu P, Phoon KM, Asopa V, Sochart DH, Kader DF (2022). The Oxford Knee Score is a reliable predictor of patients in a health state worse than death and awaiting total knee arthroplasty. Arthroplasty.

[20] Aburuz S, Bulatova N, Twalbeh M, Gazawi M (2009). The validity and reliability of the Arabic version of the EQ-5D: a study from Jordan. Ann Saudi Med.

[21] Litchfield I, Greenfield S, Turner GM, Finnikin S, Calvert MJ (2021). Implementing PROMs in routine clinical care: a qualitative exploration of GP perspectives. BJGP Open.

[22] Hariri BA, Albagmi FM, Aljaffary AA (2024). Conceptualization and establishment of value-based healthcare in Saudi Arabia: a scoping review. J Taibah Univ Med Sci.

[23] Groenewegen A, van Muilekom MM, Sieben CH (2024). The Amsterdam PROM implementation strategy: policy and pathway. NEJM Catal Innov Care Deliv.

[24] (2007). World Health Organization (WHO). Everybody's business - strengthening health systems to improve health outcomes. WHO's framework for action. https://www.who.int/publications/i/item/everybody-s-business----strengthening-health-systems-to-improve-health-outcomes.

[25] Kolukısa Tarhan A, Garousi V, Turetken O, Söylemez M, Garossi S (2020). Maturity assessment and maturity models in health care: a multivocal literature review. Digit Health.

[26] Ernst SK, Steinbeck V, Busse R, Pross C (2022). Toward system-wide implementation of patient-reported outcome measures: a framework for countries, states, and regions. Value Health.

[27] (2025). Ministry of Health. Saudi Digital Health Strategy. https://www.moh.gov.sa/Ministry/vro/eHealth/Documents/MoH-Digital-Health-Strategy-Update.pdf.

[28] Alzghaibi HA (2023). An examination of large-scale electronic health records implementation in Primary Healthcare Centers in Saudi Arabia: a qualitative study. Front Public Health.

[29] Chishtie J, Sapiro N, Wiebe N (2023). Use of epic electronic health record system for health care research: scoping review. J Med Internet Res.

[30] Stover AM, Haverman L, van Oers HA, Greenhalgh J, Potter CM (2021). Using an implementation science approach to implement and evaluate patient-reported outcome measures (PROM) initiatives in routine care settings. Qual Life Res.

[31] Al-Rossais AA, Sayeed S, Khan MS, Jaber AA, Al-Qahtani MA, Fardan AB (2020). Previous involvement in research and knowledge regarding basic research methods among doctors working at primary care in central region, Saudi Arabia. Mater Sociomed.

[32] Howsawi AA, Althageel MF, Mohaideen NK (2020). Application of the Kano model to determine quality attributes of patient's care at the primary healthcare centers of the Ministry of Health in Saudi Arabia, 2019. J Family Community Med.

[33] Fanous N, Samarkandi L, Al‐Bsheish M, Abu‐Elenin MM (2024). Patient‐reported outcomes in the Kingdom of Saudi Arabia: an insight for a healthcare system undergoing reform. World Med Health Policy.

[34] AlHadi AN, AlAteeq DA, Al-Sharif E (2017). An Arabic translation, reliability, and validation of Patient Health Questionnaire in a Saudi sample. Ann Gen Psychiatry.

[35] Abdalla R, Pavlova M, Groot W (2025). Barriers hindering the measurement of outcomes for value-based healthcare in Saudi Arabia and recommendations for improvement. J Health Manag.

[36] Greenhalgh J, Dalkin S, Gooding K (2017). Functionality and feedback: a realist synthesis of the collation, interpretation and utilisation of patient-reported outcome measures data to improve patient care. Health Services and Delivery Research.

[37] (2022). Council of Health Insurance (CHI). Value-based health care in Saudi health insurance market. White paper on value-based payment. https://www.chi.gov.sa/knowledgecenter/DocLib3/VBHC%20White%20Paper%20Version%20Final.pdf.

[38] (2025). Interoperability and integration. National Health Information Center. Riyadh.

[39] (2019). NHS Education for Scotland. Patient reported outcome measures toolkit. https://learn.nes.nhs.scot/78205/realistic-medicine/personalised-approach-to-care/patient-reported-outcome-measures-a-toolkit-to-measure-outcomes-that-matter/patient-reported-outcome-measures-toolkit.

[40] (2025). Definition of value in health in Saudi Arabia. https://cvalue.sa/publication/definition-of-value-in-health-in-saudi-arabia/.

[41] Hariri BA, Albagmi FM, Aljaffary AA (2025). Navigating the transition to value-based healthcare in Saudi Arabia: a qualitative study of policy-maker perspectives. Value Health Reg Issues.

